# *Macrodinychus* mites as parasitoids of invasive ants: an overlooked parasitic association

**DOI:** 10.1038/srep29995

**Published:** 2016-07-21

**Authors:** Jean-Paul Lachaud, Hans Klompen, Gabriela Pérez-Lachaud

**Affiliations:** 1 Departamento de Conservación de la Biodiversidad, El Colegio de la Frontera Sur (ECOSUR), Avenida Centenario Km 5.5, Chetumal 77014, Quintana Roo, Mexico; 2 Centre de Recherches sur la Cognition Animale (CRCA), Centre de Biologie Intégrative (CBI), Université de Toulouse, CNRS, UPS, 118 route de Narbonne, 31062 Toulouse Cedex 09, France; 3 Department of Evolution, Ecology and Organismal Biology, Ohio State University, Columbus OH 43212, USA

## Abstract

Mites are frequent ant symbionts, yet the exact nature of their interactions with their hosts is poorly known. Generally, myrmecophilous mites show adaptations for dispersal through phoresis, but species that lack such an adaptation may have evolved unusual specialized relationships with their hosts. The immature stages of *Macrodinychus multispinosus* develop as ectoparasitoids of pupae of the invasive ant *Paratrechina longicornis*. Feeding stages show regressed locomotor appendages. These mites complete their development on a single host, sucking all of its body content and therefore killing it. Locally high proportions of parasitized host pupae suggest that *M. multispinosus* could serve as a biological control agent. This is the ninth species of *Macrodinychus* reported as ant parasite, and the third known as parasitoid of invasive ants, confirming a unique habit in the evolution of mite feeding strategies and suggesting that the entire genus might be parasitic on ants. Several mites’ characteristics, such as their protective morphology, possible viviparity, lack of a specialized stage for phoretic dispersal, and low host specificity, combined with both the general low aggressiveness of invasive *P. longicornis* towards other ants and its possible susceptibility to generalist ectoparasites would account for the host shift in native macrodinychid mites.

In its broadest and original meaning, the term “symbiosis” refers to different organisms that live together^1^, so parasites and parasitoids are symbionts that reduce the fitness of their individual hosts or host colony. Parasites generally do not kill their hosts (except during strong infestations) and may successively attack various host individuals. In contrast, immature parasitoids develop at the expense of a single host individual before ultimately killing or sterilizing it, while adult parasitoids are free-living^2^,^3^. Mites (Arachnida: Acari) are among the most frequent symbionts of social insects in general and of ants in particular, but they are often overlooked inhabitants in their host nests^4^. Despite their astounding abundance and diversity at the level of a single host genus^5^, a single host species^6^,^7^, or even a single host colony^8^, mites are among the least studied myrmecophiles (i.e., organisms that live with ants).

At least 20 of the 109 families of the mite order Mesostigmata (Parasitiformes) are exclusively or frequently associated with ants^9^,^10^. Within this group, members of the infraorder Uropodina, a widely distributed group represented by nearly 2,300 species^10^, are the best studied. Many uropodine mites have established close symbiotic associations with social insects, especially ants^11^,^12^, although the intimacy of the relationship with their hosts and the exact nature of their feeding habits are still largely unknown. In most cases, they have been considered as scavengers feeding on fungus and bacteria–infected debris, as kleptoparasites that are occasionally able to solicit food from their hosts, or as facultative predators of other arthropods present in the host nest^4^,^11^. Generally, they do not adversely affect the ecology of the host, and only some species, such as *Trichocylliba comata* (Leonardi), which pierces the integument and sucks the haemolymph of the formicine ant *Lasius mixtus* (Nylander)^13^, can be considered ectoparasites and, sometimes, predators. However, in only two cases of the genus *Macrodinychus* Berlese, a pan-tropical taxon consisting of 23 valid species^14^, mites have been proved to act as true ant parasitoids^12^,^15^,^16^.

Various species of *Macrodinychus* have been reported from ant nests including three species native to the New World: *M. parallelepipedus* (Berlese) from Argentina, Brazil, Paraguay, and Suriname, which is associated with the dolichoderine *Dorymyrmex steigeri* Santschi^17^; *M. sellnicki* (Hirschmann & Zirngiebl-Nicol) from Trinidad, Saint Lucia, the Dominican Republic, Costa Rica, Colombia and Suriname, which is associated with the invasive formicine *Nylanderia fulva* (Mayr)^15^; and *M. multispinosus* Sellnick from some Caribbean Islands (St Kitts, Trinidad, Barbados, and Cuba)^18^, which is also found in association with *N. fulva* in Colombia^12^,^19^. Three other species have been found with ants in Japan: *M. yonakuniensis* Hiramatsu in association with the myrmicine *Pheidole megacephala* (Fabricius)^16^, and two undocumented cases of mites, which are apparently close to *M. yonakuniensis*, found parasitizing the pupae of the ponerines *Leptogenys confucii* Forel and *Brachyponera luteipes* (Mayr)^20^. Of all of these species, only the biology of *M. sellnicki* is fully known. In Colombia, immature stages of *M. sellnicki* develop as ectoparasites on the pupae of the yellow crazy ant *N. fulva*^12^,^15^. Only one host is needed for the mite to complete development and, upon moulting, the adult leaves the host pupa, which is void of all content, and continues its life as a free-living organism. This was the first ever recorded case of a mite with a parasitoid life style. Although *Macrodinychus* species have most often been collected from soil and litter habitats away from ant nests^12^, forty years ago, Hirschmann^21^ believed that myrmecophily was probably the modus vivendi for the entire genus and suggested that all of the species in this genus might be parasitic on their ant hosts, but he lacked any explicit solid evidence.

Here, we report on a new case of parasitoidism in a *Macrodinychus* species at the expense of a Mexican population of another invasive ant, *Paratrechina longicornis* (Latreille), and we review the available evidence supporting Hirschmann’s hypothesis. We further discuss the evolution of parasitoidism in *Macrodinychus* mites and their particular association with invasive ant species. Combined with evidence from the literature and the web, our results led us to conclude that parasitoidism may be a more common feeding strategy in Uropodina than previously thought. Moreover, such parasitoids could be of potential value in biological control programmes against some invasive ants.

## Results

### Ant sample composition and prevalence of parasitism

**Figure 1:**
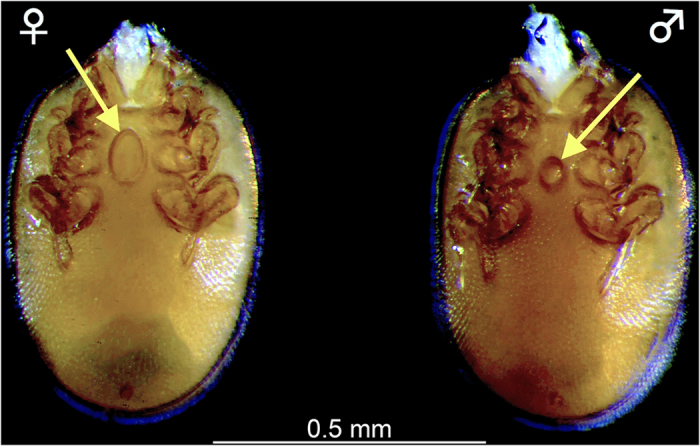
*Macrodinychus multispinosus* Sellnick adults. Left: ventral view of a female; right: ventral view of a male. The genital shield (yellow arrows) presents a marked sexual dimorphism: the male has a sub-circular small genital plate compared with that of the female, which is oval and larger. Photo: H. Bahena Basave.

In addition to an initial sample of *P. longicornis* from Chetumal (Quintana Roo, Mexico) (see Methods), five samples from Laguna Guerrero and eleven from Mahahual (both sites also located in Quintana Roo) were obtained. The proportion of ant brood varied between samples and among seasons (Table 1), but pupae (the main host stage target of the mites, see below) were present at all times. A total of 1,630 mites were recovered from the 6,210 available ant host pupae. Most mites were deutonymphs (see Table S1) but a few were well-developed, teneral adults and could be sexed (27 females, 19 males; n = 46, see Fig. 1). Only 10 larvae could be retrieved, but their small size at this stage made them difficult to see.

Eight of the 17 sampled *P. longicornis* colonies (47.1%) were parasitized; parasitism rates ranged between 0.4% and 76.8%, with a median of 7.0% (Table 1), and a global proportion of parasitized pupae of 26.2%. Colony sample size was strongly associated with both the number of parasitized pupae (large colony samples had more parasitized pupae; Permutation test, P = 0.01439) and with parasitism rates (large colony samples were more susceptible to mite attack; GLM adjusted for overdispersion (χ^2^_1_ = 6.38, P = 0.01). In addition, parasitism rates differed significantly both between sites (GLM adjusted for overdispersion: χ^2^_1_ = 76.5, P < 0.01) and between seasons (χ^2^_1_ = 25.9, P < 0.01), but there was no interaction between seasons and sites (χ^2^_1_ = 0.008, n.s.). In January, during the dry season, no mites were found at Mahahual where *P. longicornis* nests in coconut tree palms, but they were sparsely present (only two infested colonies with parasitism rates <1%) in September, during the rainy season. In contrast, at Laguna Guerrero, where colonies nested under stones, parasitized pupae were observed in all samples and during all seasons (Table 1). Coconut trees were present at this site, but unlike at Mahahual, *P. longicornis* was never found nesting in the palms.

### Biology of the parasitoid mite

The mite specimens run to *Macrodinychus multispinosus* in the taxonomic key of Kontschán^14^, and the revised material matches the original description of the adults by Sellnick^18^ (see Fig. S1). The samples from this study were also compared with a *M. multispinosus* specimen from Colombia in the OSAL Collection (OSAL 0046554)^19^.

**Figure 2:**
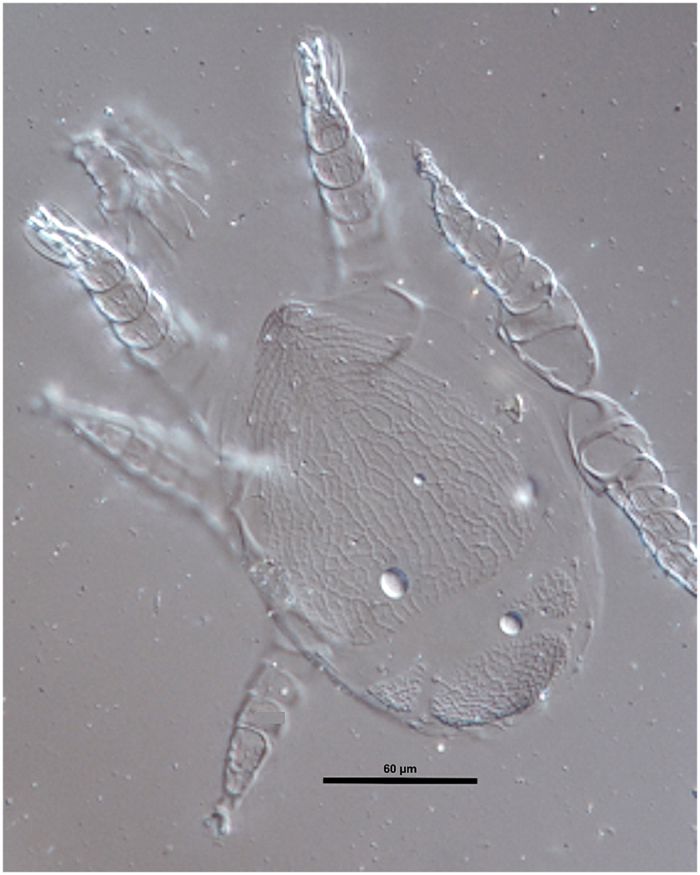
*Macrodinychus multispinosus* Sellnick larva. Dorsal view of a slide-mounted specimen (OSAL 0102204) found attached to a *Paratrechina longicornis* pupa. Magnification 400x, differential interference contrast illumination. Photo: H. Klompen.

**Figure 3:**
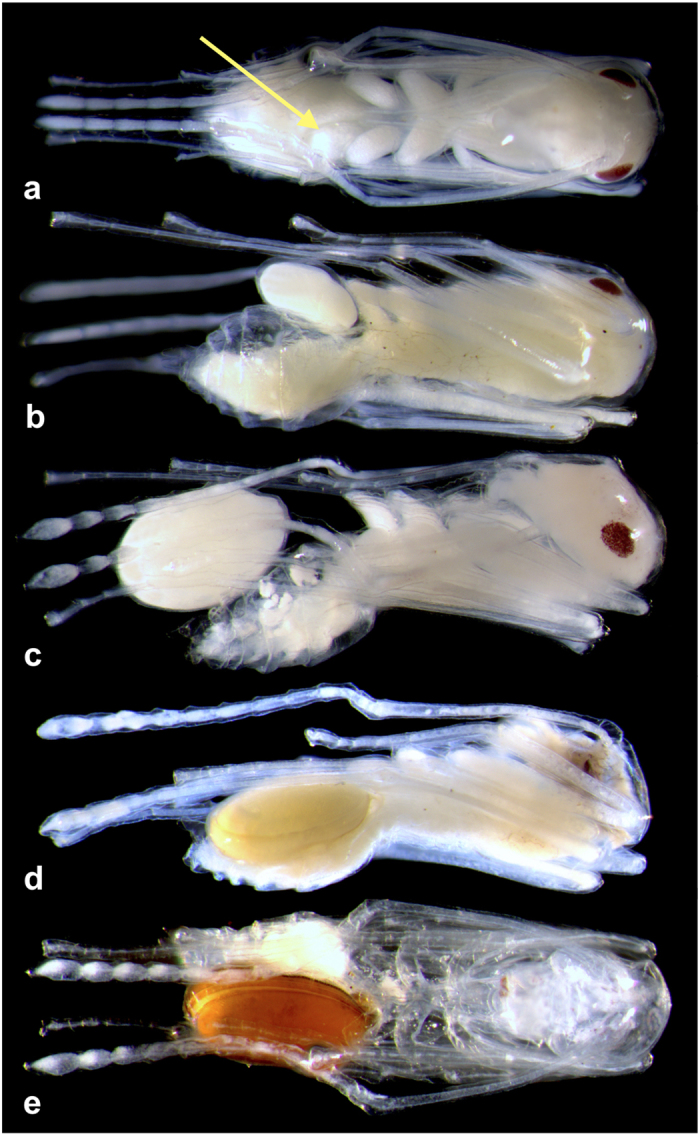
*Macrodinychus multispinosus* developmental stages and progressive morphological changes of the ant host pupa. (**a**) Feeding mite larva anchored (see arrow) on the underside of the third left coxa of a worker pupa; (**b**) mite protonymph on half-depleted ant host gaster; (**c**) mite deutonymph on an almost empty host gaster; (**d**) host gaster totally occupied by the fully grown deutonymph; (**e**) teneral pigmented adult mite on fully drained, unviable host worker pupa. Photos: H. Bahena Basave.

**Figure 4:**
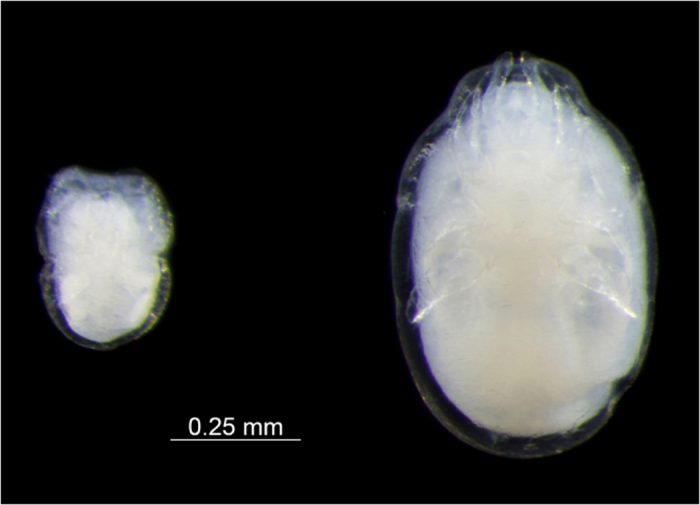
Motionless, specialized feeding stages of *Macrodinychus multispinosus*. Left: ventral view of a protonymph; right: ventral view of a deutonymph. Note the very short, non-functional legs. The mites have been separated from their *Paratrechina longicornis* hosts. Photo: H. Bahena Basave.

**Figure 5:**
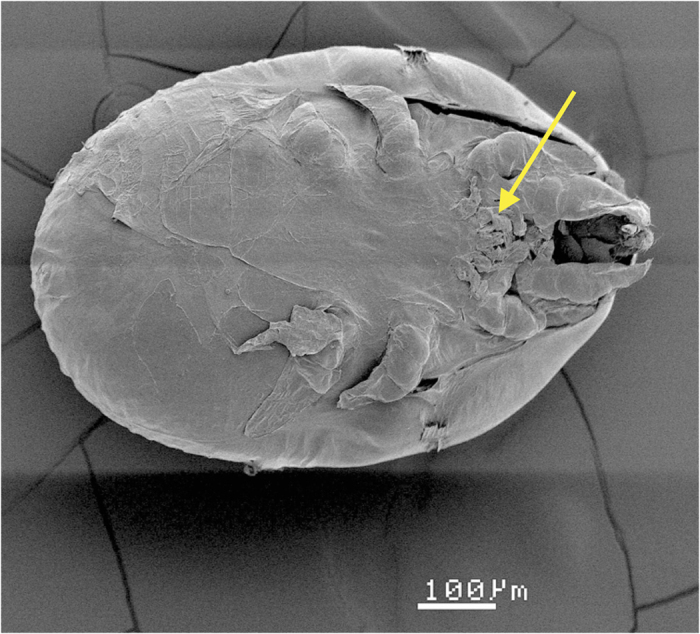
Ventral view of a *Macrodinychus multispinosus* deutonymph. Scanning electron micrograph. Exuviae of the previous developmental stages remain attached to the ventral side of the developing mite (see arrow). Note the very short, non-functional legs of the deutonymph. Photo: G. Nieto.

*Macrodinychus multispinosus* undergoes three moults: from larva to protonymph to deutonymph to adult. Larvae measure 174.2 ± 7.8 × 104.2 ± 7.9 μm (length x width; mean ± SD, n = 10), possess long legs relative to their body (Fig. 2) and are mobile. The length of legs II was 84.2 ± 7.6 μm on average (n = 10), i.e., approximately 48.3% of the body length. The larvae attach to the underside of the third coxae of their host ant pupae, between the thorax and the gaster, and small larvae were only observed attached to newly transformed ant pupae, i.e., those without eye pigmentation. Subsequent to attachment, mite larvae stay motionless and begin consuming the host tissues by sucking. The early developmental stages of the mite (feeding larvae and young protonymphs) are, most often, difficult to see and may even be confounded with an apparent supplemental coxa (see Fig. 3a). During development, individual mites remain anchored at the place of their original attachment and maintain their position on the ant host (Fig. 3b). As in *M. sellnicki*, both nymphal instars are inactive, show regression in their locomotor appendages (legs II of the deutonymph measure 218 μm, i.e., approximately 29% of the body length, and are not functional (Figs 4 and 5), but they gain considerable body mass as development proceeds (Fig. 3b–e). On average, protonymphs measured 480 ± 50 × 350 ± 30 μm (length x width; n = 34), and deutonymphs measured 750 ± 80 × 500 ± 50 μm (n = 76). Adult males measured 780 ± 20 × 520 ± 20 μm and females were 800 ± 20 × 520 ± 20 μm. Males and females differed significantly in body length (two-tailed Student’s t-test: t_28_ = 3.59, P = 0.001, n_1_ = n _2_ = 15) but not in width (two-tailed Student’s t-test: t_28_ = 0.53, P = 0.6, n_1_ = n _2_ = 15).

In terms of deutonymph anatomy, tarsi I resemble those in *M. sellnicki*^12^ with a single, large, spine-like seta and a minimum of 18 additional tiny sensilla; tarsi II carry at least 3–5 small sensilla each. Legs I-II do not appear to retain any tibial, genual, or femoral setae. Coxae I have strongly developed anteroventral and posteroventral setae, again as in *M. sellnicki*; sternal setae *st1* were not observed. The position of the deutonymph mouthparts did not allow for any observations of the shape of the chelicera or gnathosoma.

As the mite grows, a concavity is formed at the place of attachment that secures its position on the host (Fig. 3c). Fully grown deutonymphs occupy the entire gaster of their host pupa, which is reduced to its fine exoskeleton. At this stage of the development of the parasitoid, parasitized ant pupae appear to have a normal gaster, although the tip is more rounded (Fig. 3d). However, they can be easily identified by external changes due to mite feeding, such as the partial loss of eye pigmentation (but see Fig. 3a–c) and, above all, the slow but constant diminution of body bulk as the host tissues are progressively sucked out by the mite, and the host pupa becomes transparent and finally shrivels (Fig. 3b–e). The exuviae of the precedent instars remain attached to the deutonymph (see Fig. S2), and the deutonymphal envelope is retained until adult eclosion. Parasitized ant pupae continue to be tended by workers and are picked up and transported when the nest is disturbed. In the alcohol-preserved material, several ants were found to be carrying parasitized pupae in their mandibles (see Fig. S3).

In general, only one mite develops on a single host although three cases of superparasitism involving attack by two mite larvae were recorded (less than 0.2% of all parasitized host pupae), and in one of the cases, both mites developed to at least the deutonymphal stage (see Fig. S4) before being collected and preserved in alcohol. Worker pupae seem to be the principal target of the mites, although during the production of sexual individuals, a small fraction of the males were found to be parasitized; three male *P. longicornis* pupae out of 46 (6.5%) were parasitized while no female pupae were parasitized out of seven collected (Table 1 and Table S1). All of the other 1,627 parasitoid mites were obtained from 6,157 worker pupae (26.4%).

## Discussion

Mite-ant interactions are numerous and diverse and range from facultative tolerance in ant nests to very specific symbiotic interactions. For example, in the obligate myrmecophile *Aribates javensis* Aoki *et al*.^22^ and in the less-specialized myrmecophile *Protoribates myrmecophilus* Aoki & Ito^23^ (Acariformes: Oribatida), the *Myrmecina* sp. hosts feed and even carry the mites when moving to a new nest site. Mesostigmata mites and social insects have a long evolutionary history and phoretic relationships with their ant hosts apparently date back from the Eocene, almost 50 Myr^24^. More specifically, uropodine mites are commonly known for their phoretic habits with deutonymphs representing a specialized life stage with morphological and physiological adaptations for dispersal through phoresy; for example, many species are able to produce an anal pedicel for temporary attachment to a carrier^25^,^26^,^27^.

Although ants have been intensively studied and collected, only two documented records of uropodine mites with a parasitoid life style have been reported to date. Both records involve native mite species of the macrodinychid genus *Macrodinychus* parasitizing newly introduced populations of tramp ant species. The first record concerns *M. sellnicki*, which is probably native to Colombia^14^, parasitizing the pupae of *N. fulva*, an invasive agricultural pest in sugar cane in Colombia originating from east-central South America (southern Brazil, Paraguay and northeastern Argentina)^28^, with parasitism rates of up to 90% in some colonies^15^. The second record concerns *M. yonakuniensis*, which is native to Japan^14^ and parasitizes, in Okinawa, the pupae of a recently introduced population of the big headed ant *P. megacephala*, believed to be native to Africa^29^, with 92% of the nests and 15.5% of all pupae being parasitized^16^. Here, we report on a third species of uropodine mite in the same genus, *Macrodinychus*, acting as a true primary parasitoid of another invasive ant, *P. longicornis*, with up to 47.1% of the colonies infested and 26.2% of all pupae parasitized, and we provide the first documented life history description of this parasitic species. We also provide some details that may serve to answer basic questions related with the biological and ecology-related characteristics of tramp ant species that make them suitable hosts for *Macrodinychus* mites, and that could explain the establishment of such new parasitic associations in areas of invasion. Surprisingly, the three macrodinychid species with a parasitoid lifestyle that have been documented so far lack any specialized stages for phoretic dispersal and are thus atypical among the uropodine mites, showing regressed, inactive proto- and deutonymphs that specialize in sucking haemolymph and other cell tissues from their host. Notably, phoretic deutonymphs are also absent in three other ant-associated uropodine families, Oplitidae, Trachyuropodidae and Trichocyllibidae *s.l*. However, in these taxa, the adults are found on the host, and the ecology of the immature stages is essentially unknown.

*Macrodinychus multispinosus* was described by Sellnick^18^ from a hundred males and females collected among plant decay, but its biology and life cycle were hitherto unknown. Its life history follows the general life cycle outlined for *M. sellnicki*^15^. The young larvae are responsible for host searching, and both proto- and deutonymphs are motionless, specialized feeding stages. The attachment site of the larvae on their host seems to be species-specific; *M. sellnicki* larvae attach in the gular region on the underside of the head capsule of the host pupa^12^,^15^, while *M. yonakuniensis*^16^ and *M. multispinosus* attach to the gaster, in the ventral region underneath one of the third coxae.

Not all of the host castes seem to be equally attacked by the parasitoid mites. *Macrodinychus sellnicki* parasitized any caste of *N. fulva*, although parasitism on queen pupae was infrequent^15^, and in the case of *P. megacephala*, the prevalence of parasitism varied strongly among castes with soldier and male pupae being the most targeted^16^. In *M. multispinosus*, the target host seems to be the worker caste of *P. longicornis* with 26.4% of all worker pupae being parasitized, although some male pupae were also parasitized (only 6.5% of the available male pupae). None of the seven female pupae that were collected were parasitized, but the very low number of sexual pupae (especially, of female pupae) in our samples currently prevents reaching any conclusion as to the preferred host caste targeted by *M. multispinosus*.

The prevalence and virulence of *M. multispinosus* mites have both individual and colony-level fitness costs for their hosts. Mites exact a high fitness cost from *P. longicornis* at the colony level with losses due to parasitism of up to 76% of the pupae in some nests or up to 44.3% if only parasitism by deutonymph and adult mites (which have already sucked out all of the contents of their host) is considered (see Table S1). While the median parasitism rate was only 7.0%, the global proportion of *P. longicornis* pupae parasitized by *M. multispinosus* reached 26.2%; even considering only deutonymphs and adult mites, at least 16.3% of all of the examined host pupae were killed, suggesting that this mite could be of potential use in the biological control of this invasive ant species. Furthermore, our parasitism rates are likely a very conservative estimate since mites tended to fall off of the ant pupae after being fixed in alcohol, and the small larvae may have remained undetected among the similarly sized and very numerous host eggs. As in other specialized ant parasitoids^30^, mite parasitism can be highly restricted in time and space; mites were almost absent from arboreal colonies nesting in palms at Mahahual, while ground-nesting colonies at Laguna Guerrero were always parasitized although the prevalence of parasitism was highly variable. Such differentiation in the patterns in mite parasitism likely depends on numerous biotic and abiotic factors (variations in climate between the study sites, differences in the invasion date of the host ant, etc.) but also on differences in their host nesting site, which could influence host detection and location by the parasitoids and, consequently, parasitism rates^31^. As hypothesized by Michener^32^ for halictine and ceratinine bees, host nest detection and location by potential wingless parasites would be easier for ground-nesting species, which are distributed in a two-dimensional space, than for arboreal-nesting species distributed in a three-dimensional space.

The invasion success of biological invaders has often been tentatively explained by the “enemy release” hypothesis that natural enemies are not carried along with the invasive organisms in their introduced range^33^. However, the complexity of the processes underlying biological invasions requires alternative explanations because novel associations, such as with native predators or parasites, may form in the invaded range^34^,^35^. Invasive ants tend to displace native ants and become the dominant species in a habitat, and this, in turn, would select for adaptation by indigenous parasites and eventually to high parasitism pressure^36^. As a consequence, the “invasive syndrome”^37^ might predispose invasive ants to attack by generalist ectoparasites. As with many introduced populations of other invasive ant species, the populations of the tramp species associated with *Macrodinychus* mites are unicolonial, polygynous, omnivorous, and generally lack intraspecific aggression, which are all traits that promote invasiveness and contribute to their success in introduced areas^36^,^37^,^38^. Apart from competition with resident species for food resources, invasive species interact with indigenous natural enemies or with mutualists that may facilitate establishment and spread in new habitats^39^,^40^. In the early stages of spread, invasive ant species interact with native fauna and can eventually adopt native generalist myrmecophiles as occurred in *Lasius neglec*tus Van Loon *et al*., an invasive ant in Europe^41^,^42^, and in the Argentine ant *Linepithema humile* (Mayr) in Spain^41^. Unlike other invasive ant species, *P. longicornis* is neither territorial nor aggressive towards other ants^43^, a trait that may have facilitated mite host shift and integration. It is noteworthy that none of the three known parasitoid macrodinychid mites appear to be host specific. Even though *M. sellnicki* showed a clear preference for *N. fulva*, it was not exclusive to this species; in the two Colombian localities where it was parasitizing *N. fulva*, *M. sellnicki* also attacked the pupae of the native *Solenopsis geminata* (Fabricius) with a parasitism prevalence of 2.5 and 30%, respectively (*vs.* 28 and 44%, for *N. fulva*)^15^. Similarly, *M. yonakuniensis* in addition to attacking *P. megacephala* in Okinawa, also parasitized the native *P. noda* Smith^16^, and *M. multispinosus* has been found in association with another host, *N. fulva*, in Colombia^12^,^19^. For *M. sellnicki* and *M. yonakuniensis*, it has been suggested^15^,^16^ that a host shift would have occurred after the introduction of *N. fulva* into Colombia and of *P. megacephala* into Japan, respectively, and the same process is likely to have occurred in populations of *P. megacephala* introduced to New Caledonia that are also currently parasitized by *M. yonakuniensis* (Julien Le Breton, personal communication). Given that *M. multispinosus* is known from the Caribbean Islands (St Kitts, Trinidad, Barbados, and Cuba)^14^ and now from southern Mexico, all of which are localities where *P. longicornis* has also been reported^44^, it is likely that this mite might also have host shifted when *P. longicornis* was introduced. However, this leaves the question open as to the native host of *M. multispinosus* in southern Mexico. Despite extensive surveys in Laguna Guerrero (J.-P.L. & G.P.-L., unpublished data) we have failed to discover any other ant species that are parasitized by this mite. However, proto- and deutonymphs of *M. multispinosus* appear as specialized stages for living as parasites because they are not mobile. Therefore, they are not likely to survive freely and we have to assume that even though the association with *P. longicornis* might be only occasional and the result of an opportunistic strategy, a native host does exist, but still remains to be discovered.

Apart from the possible susceptibility of *P. longicornis* to attack by native generalist ectoparasites, several morphological and life history traits exhibited by macrodinychid mites might have facilitated the transition to parasitoidism and allowed adults to thrive unmolested within ant nests. First, *Macrodinychus* larvae possess strongly developed claws that allow mites to firmly attach to their host, a characteristic that is likely related to living as an ectoparasite^12^. Second, the typical morphology of the Uropodidae (commonly known as tortoise mites) may have facilitated their irruption and later integration into ant colonies. Adult uropodines have a hard sclerotized cuticle and their short legs can be withdrawn into ventral depressions (pedofossae) (see Supplementary Fig. S1). Furthermore, the idiosomal margin is curved and may both help maintain the developing mites in place and somewhat protect the adults from ant attacks. For example, when *N. fulva* workers tried to grasp *M. sellnicki* adults in artificial nests, the smooth and hard cuticle of the mites thwarted their attack, so the ants tended to ignore the adult mites^12^. The resemblance of the deutonymph to the gaster of the host pupa in size and shape may afford further protection during mite development in a similar manner as has been proposed for the symbiont cricket *Myrmecophila americanus* Saussure that seems to mimic the gaster of the *P. longicornis* queen^45^. Finally, viviparity is common in several species of Uropodina^46^ including *M. sellnicki* and other species of this genus^12^, and although it has not yet been demonstrated in *M. multispinosus*, viviparity would enhance the chances of the parasitic larvae to rapidly encounter hosts that are constantly relocating their nests and/or enable the mites to maintain their attachment if the brood is relocated^12^ or when colonies multiply by colony fission as is the case for *P. longicornis*^47^.

The relationships between *Macrodinychus* spp. parasitoids and their tramp ant hosts represent a unique case in the evolution of mite feeding strategies in the Mesostigmata as well as the entire Acari^12^. Considering both the approximately 16,000 valid ant species^48^ and the more than 48,000 mite species^49^, it is unlikely that parasitoidism would have only evolved thrice in the Acari and only in the single genus *Macrodinychus*. Currently, 23 *Macrodinychus* species are known from all over the world^14^, of which only the biology of *M. sellnicki*^15^ and *M. multispinosus* (this study) are fully known. Including the two undocumented cases reported in Japan^20^, which probably involve two new species close to *M. yonakuniensis*, 4–6 species of *Macrodinychus* belonging to three subgenera (*Macrodinychus*, *Bregetovamacrodinychus*, and *Loksamacrodinychus*) have already been reported as parasitic on ants. A re-examination of material deposited in the collection of the Acarology Laboratory of Ohio State University, revealed two additional cases of such associations. One (collected by L. Sekerka: OSAL 01044624-625) involved a male and a deutonymph of *M. mahunkai* Hirschmann and the ecitonine ant *Labidus coecus* (Latreille) in Ecuador. The other (collected by C. von Beeren: OSAL 0103942-944 and OSAL 0106706-711) referred to multiple specimens (adult and nymphs) of *M. extremicus* Kontschán (belonging to the fourth subgenus, *Monomacrodinychus*) associated with the ponerine ant *Leptogenys processionalis distinguenda* (Emery) in Malaysia. Finally, a male of *M. shibai* Hiramatsu (also belonging to *Monomacrodinychus*) still in its deutonymphal cuticle, was collected from the Philippines (collected by B. Gerdeman and R. Garcia) in association with unidentified, and unfortunately not conserved, ant specimens. Further work on the taxonomy and parasitic status of the other species of the genus is needed to determine whether Hirschmann’s hypothesis^21^ can really hold for the entire genus. However, it is notable that 7–9 of the 23–25 species of *Macrodinychus*, including representatives of all four subgenera^14^, have now been reported to be parasitic on ants, and that at least three of them are true parasitoids. Moreover, many more instances of such parasitoid mites might certainly be discovered in the future.

## Methods

### The ant host

The longhorn crazy ant, *P. longicornis*, is a globally distributed invasive pest ant that is common in highly disturbed and anthropogenic environments of the tropics and subtropics as well as in temperate areas^44^. Due to its rapidly expanding range, explosive localized population growth, resistance to control, and its significant effect on the population growth of phloem-feeding agricultural pests, *P. longicornis* has emerged as a serious pest problem in tropical and subtropical areas^50^,^51^. As a consequence, this species has been the focus of several and very diverse studies in recent years. Workers exploit honeydew-producing Hemiptera and are also effective hunters and scavengers^43^ employing multiple specialized recruitment and foraging strategies^52^,^53^ and optimal cooperative transport^54^. Such characteristics, along with an unusual mode of reproduction that allows *P. longicornis* colonies to maintain heterozygosity over generations despite population bottlenecks and sib mating^47^, could explain its success as an invasive organism^47^,^53^,^54^.

Intensive collecting efforts focused on the nests of this species, both in and out of its presumed native range, have demonstrated associations with a small number of species-specific myrmecophilous arthropods^44^,^45^ and a diverse community of Actinobacteria and soil-borne saprophytic as well as insect-pathogenic microfungi^55^,^56^. However, compared with other non-invasive ant groups^3^, the number of organisms associated with this tramp species (i.e., ant species that have been inadvertently spread around the world by human commerce^38^) is very low, and no association with any mite species has previously been reported.

### Sampling and statistical analyses

An initial, fortuitous, collection of several *P. longicornis* workers along with brood and two queens during a colony emigration at an urban site in Chetumal (Othon P. Blanco Municipality, Quintana Roo: 18°30′29″N, 88°18′57″W; 8 m above sea level (asl)) in November 2014 yielded three ant pupae being parasitized by mites. Thereafter, various colony samples (accessible colony fragments found under stones or between the fronds of coconut palms) were collected between January and October 2015 at two sites in the Othon P. Blanco Municipality in the southern region of Quintana Roo: a coastal lagoon plot located at Laguna Guerrero (18°41′32″N, 88°15′41″W, 3 m asl), 21 km directly north of Chetumal, and a coastal beach plot located at Mahahual (18°37′57″N, 87°43′46″W, 3 m asl), 61 km directly northeast of Chetumal. Both sites have been subjected to anthropogenic changes, and patches of original vegetation (mangrove) and man-made constructions were intermixed.

*Paratrechina longicornis* colonies were located under large stones in Laguna Guerrero, while they essentially nested between the fronds of coconut palms (*Cocos nucifera* Linnaeus) in the sandy soil of Mahahual. Ants and associated fauna were collected using an entomological aspirator, and colonies were collected from under different stones or trees according to the site. All of the collected specimens were preserved in 99% ethanol, and individuals (adult ants and immature stages) were carefully examined under a stereomicroscope (Nikon SMZ745T) and counted, except for eggs, which were examined but not counted. All of the material was checked for attached mites whose position and number were noted. Mites remaining in the alcohol sediments were also counted, and the parasitism rates were adjusted to account for these specimens. Thus, the adjusted percentages given in Table 1 are the percentages of “attached + detached” specimens and may be a more realistic estimate of the actual percentages of mite parasitism. Mites were measured under a stereomicroscope with a micrometre, except for larvae which were measured under an optical microscope at 400x magnification (temporary slides with lactic acid as the mounting medium). Measurements (body length along the dorsum, not including the gnathosoma, maximum body width, and maximum legs II length from coxa to tarsus) are given in micrometres (μm) for all of the stages. The comparison of male and female body measurements was performed with Student’s t-tests. Potential factors influencing parasitism rates (site and season) were explored with a Generalized Linear Model (GLM) with a binomial response (parasitized hosts as a function of total available hosts) corrected for overdispersion. Whether large colony samples have higher infection rates was also investigated with a GLM with a binomial response and corrected for overdispersion. Finally, a permutation test (with 5000 permutations) was used to test for independence of the number of parasitized pupae and the size of the colony samples. Statistical analyses were performed in R^57^.

Some mite specimens were cleared in lactic acid and mounted in Hoyer’s medium on microscope slides for further identification. Voucher specimens of both mites and ants were deposited in the arthropod (ECO-CH-AR: AA-3331) and formicid (ECO-CH-F: F-1098–1099) collections of El Colegio de la Frontera Sur in Chetumal (Mexico), and at the Acarology Laboratory of Ohio State University in Columbus (USA) (OSAL 0102202-0102204; http://acarology.osu.edu/database).

Pictures on Figs 1 and 3 are composite of several photos taken at different levels of focus and merged using Helicon Focus (Helicon Soft Ltd).

## Additional Information

**How to cite this article**: Lachaud, J.-P. *et al*. *Macrodinychus* mites as parasitoids of invasive ants: an overlooked parasitic association. *Sci. Rep.* **6**, 29995; doi: 10.1038/srep29995 (2016).

## Supplementary Material

Supplementary Information

## Figures and Tables

**Table 1:** *Paratrechina longicornis* sample composition and percent parasitism by *Macrodinychus multispinosus* in three localities of southern Quintana Roo, Mexico.

Locality	Date	Season	Q	Alate Q	Males	Workers	Female pupae	Male pupae	Sexual larvae	Worker pupae	Worker larvae	Eggs	Parasitized host pupae	Percent parasitism
Chetumal	09/09/14	Rainy	2	0	0	28	0	0	0	13	15	+	3	23.1
Laguna Guerrero	11/01/14	Dry	0	0	0	116	0	0	0	268	140	−	5	1.9
	01/24/15	Dry	1	0	0	579	0	2	0	1594	609	+	377	23.6
	02/01/15	Dry	0	0	0	18	0	0	0	30	12	−	2	6.7
	06/07/15	Rainy	2	0	0	530	0	2	0	176	127	+++	13	7.3
	07/27/15	Rainy	1	0	20	1090	1	11	0	1585	1660	+++	1219	76.3
Mahahual	03/08/15	Dry	0	0	0	59	0	0	0	63	27	−	0	0
	03/08/15	Dry	0	0	0	43	0	0	0	22	4	−	0	0
	03/08/15	Dry	0	0	0	2	0	0	0	33	27	−	0	0
	03/08/15	Dry	0	0	0	14	0	0	0	135	111	−	0	0
	03/08/15	Dry	0	0	0	84	1	0	9	6	4	−	0	0
	09/27/15	Rainy	2	0	2	1781	1	11	10	1318	1503	+++	10	0.8
	09/27/15	Rainy	0	0	1	195	0	2	16	145	128	+	0	0
	09/27/15	Rainy	0	0	0	140	0	5	3	313	62	−	0	0
	09/27/15	Rainy	0	0	1	181	0	3	7	110	89	+	0	0
	09/27/15	Rainy	1	1	1	168	0	4	3	71	252	+	0	0
	09/27/15	Rainy	0	0	14	163	4	6	0	275	259	−	1	0.4

The presence of eggs in the nests is denoted as follows: (−) no egg; (+) a few eggs; (+++) numerous eggs.
