# Supplementary material for: Macrodinychus mites as parasitoids of invasive ants: an overlooked parasitic association
Source: Sci Rep. 2016 Jul 21;6:29995. doi: 10.1038/srep29995 (PMC4956750; doi:10.1038/srep29995)
Supplement: Supplementary Information [file srep29995-s1.pdf]

Supplementary Information

Macrodinychus mites as parasitoids of invasive ants: an overlooked parasitic association

Jean-Paul Lachaud[1,2,*], Hans Klompen[3] & Gabriela Pérez-Lachaud[1]

[1] Departamento de Conservación de la Biodiversidad, El Colegio de la Frontera Sur (ECOSUR), Avenida Centenario Km 5.5, Chetumal 77014, Quintana Roo, Mexico

[2] Centre de Recherches sur la Cognition Animale (CRCA), Centre de Biologie Intégrative (CBI), Université de Toulouse, CNRS, UPS, 118 route de Narbonne, 31062 Toulouse Cedex 09, France

[3] Department of Evolution, Ecology and Organismal Biology, Ohio State University, Columbus OH 43212, USA

Content:

Figure S1. Ventral view of a *Macrodinychus multispinosus* adult female.

Figure S2. Resilient exuviae of the previous stages of a mite male.

Figure S3. Host worker carrying a parasitized worker pupa.

Figure S4. Superparasitism by *Macrodinychus multispinosus*.

Table S1. Host targets of the different developmental stages of *Macrodinychus multispinosus*.

Figure S1. Ventral view of a *Macrodinychus multispinosus* adult female. Scanning electron micrograph. Note that legs may be withdrawn into special depressions (pedofossae). Photo: G. Nieto.

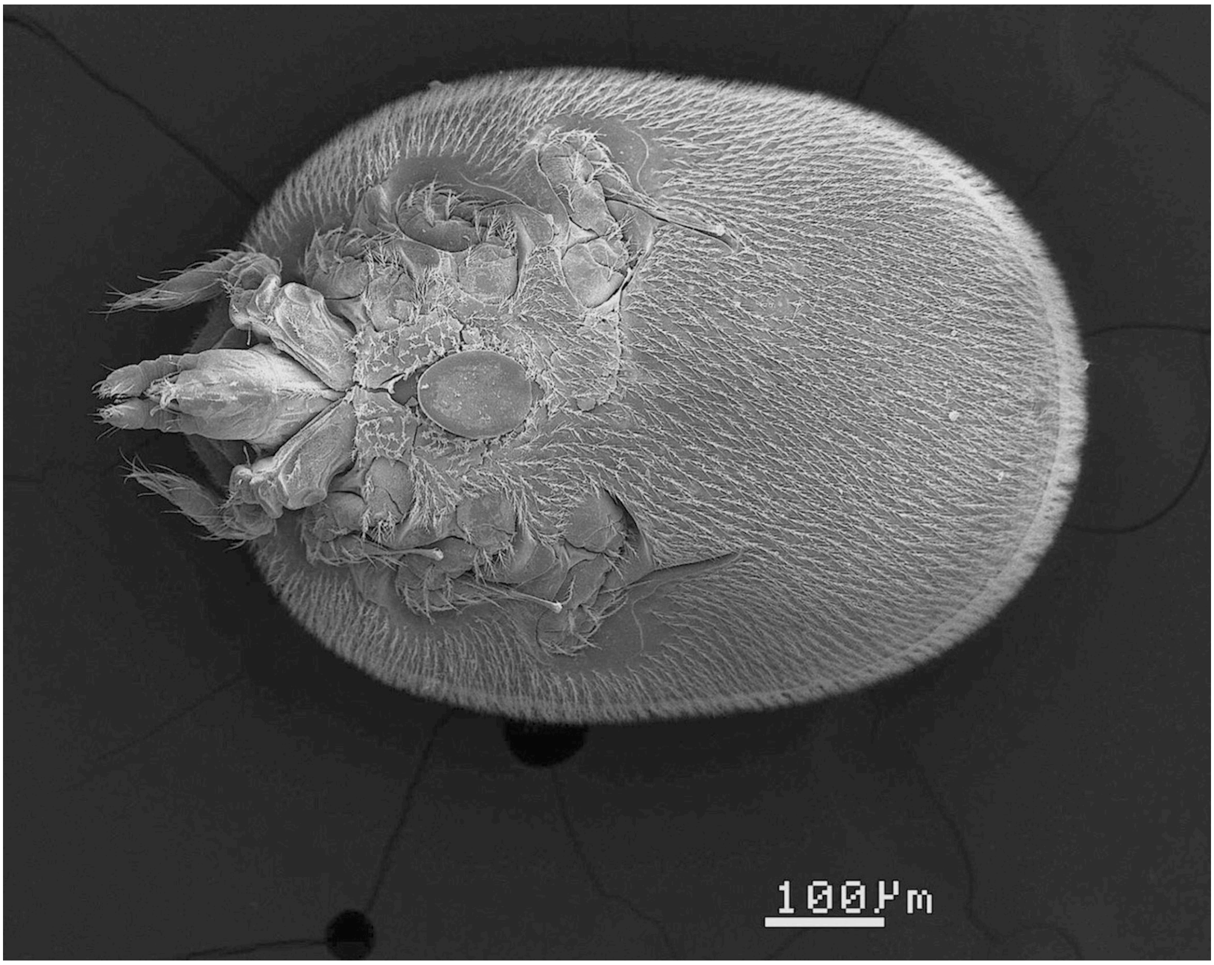

Figure S2. Resilient exuviae of the previous stages of a mite male. Slide-mounted exuviae of the immature stages of *Macrodinychus multispinosus*: cuticle of the larva (a) still preserved on the cuticle of the deutonymph (b). Photo: H. Klompen.

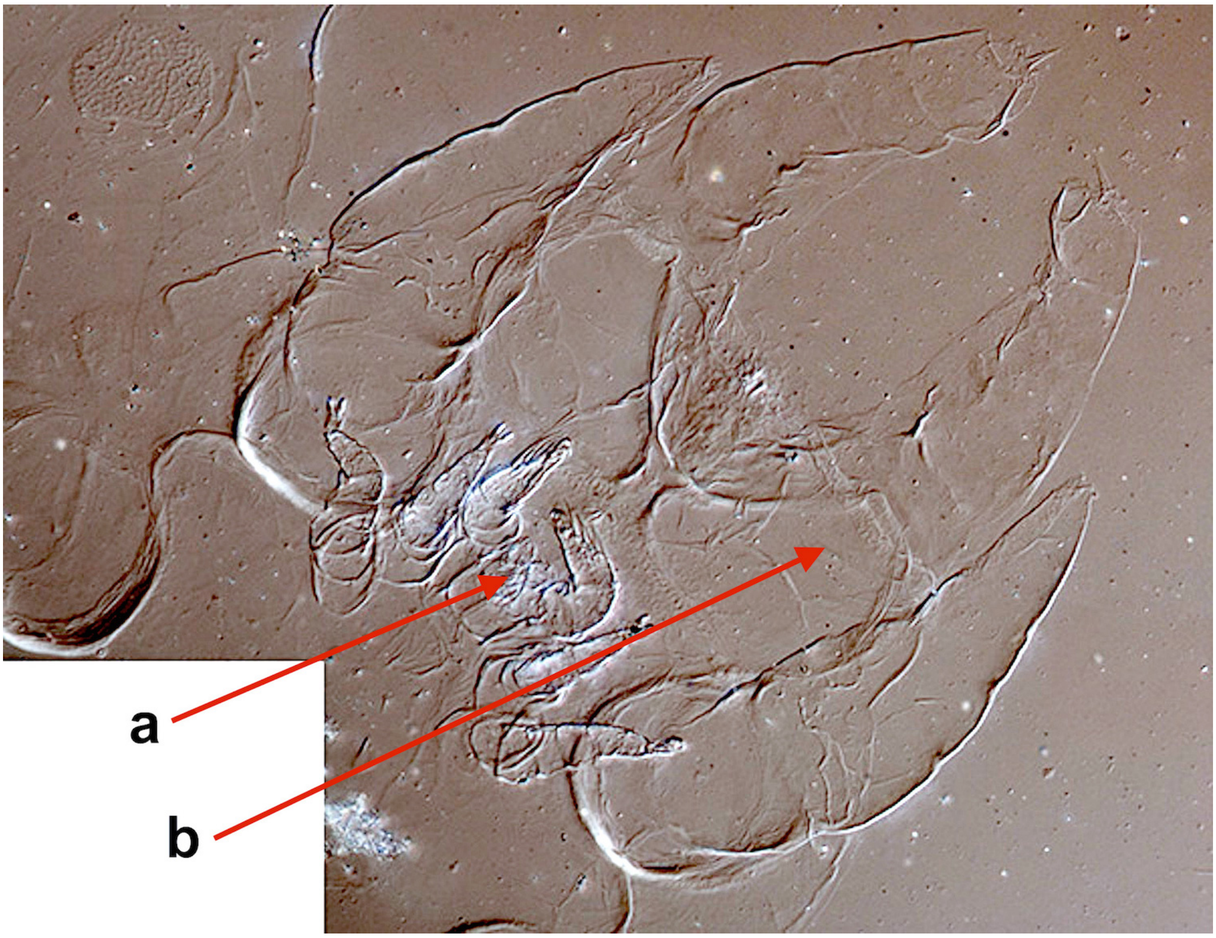

Figure S3. Host worker carrying a parasitized worker pupa. Alcohol preserved *Paratrechina longicornis* worker carrying in its mandibles a worker pupa parasitized by a teneral adult mite. Photo: G. Pérez-Lachaud.

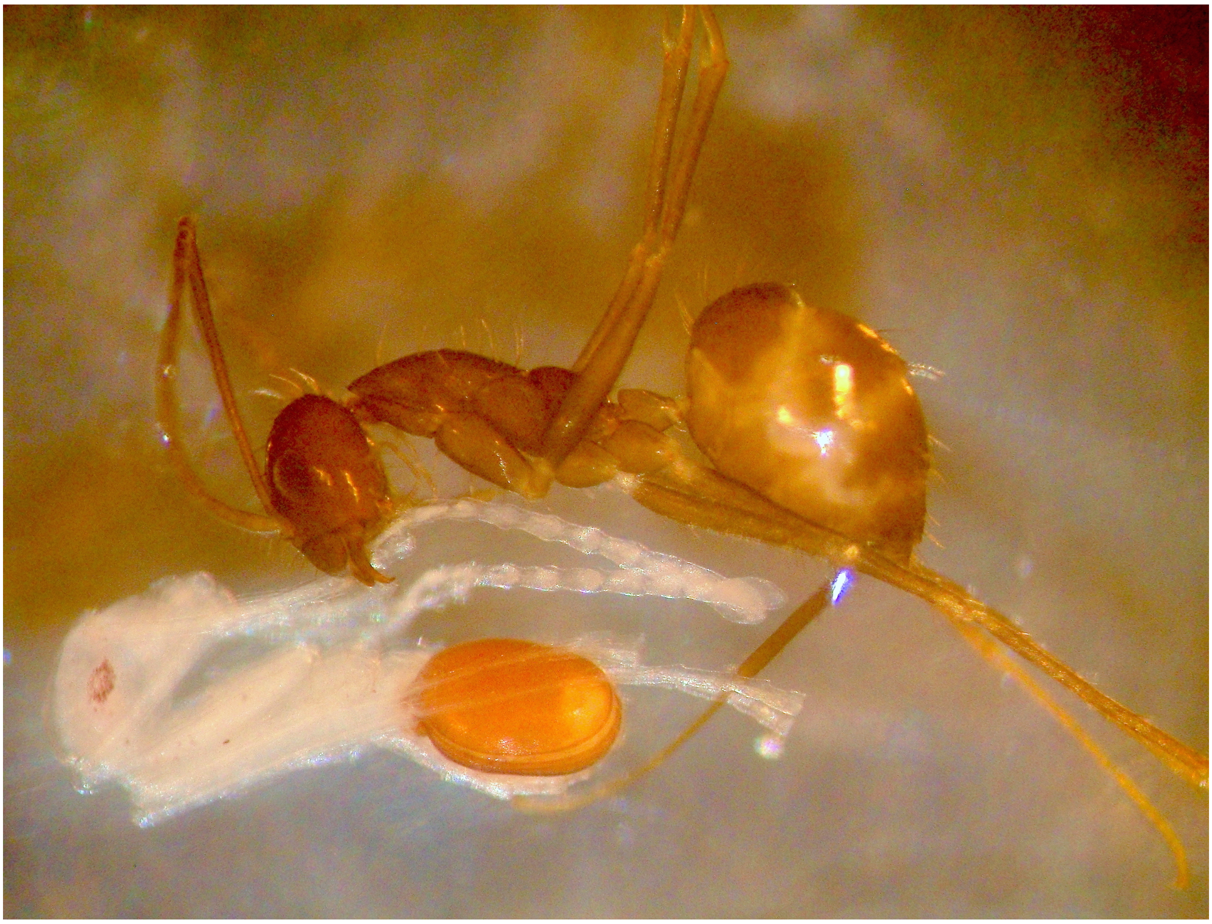

Figure S4. Superparasitism by *Macrodinychus multispinosus*. Rare example of a *Paratrechina longicornis* worker pupa parasitized by two deutonymphs (see arrows) of *M. multispinosus*. Photo: G. Pérez-Lachaud.

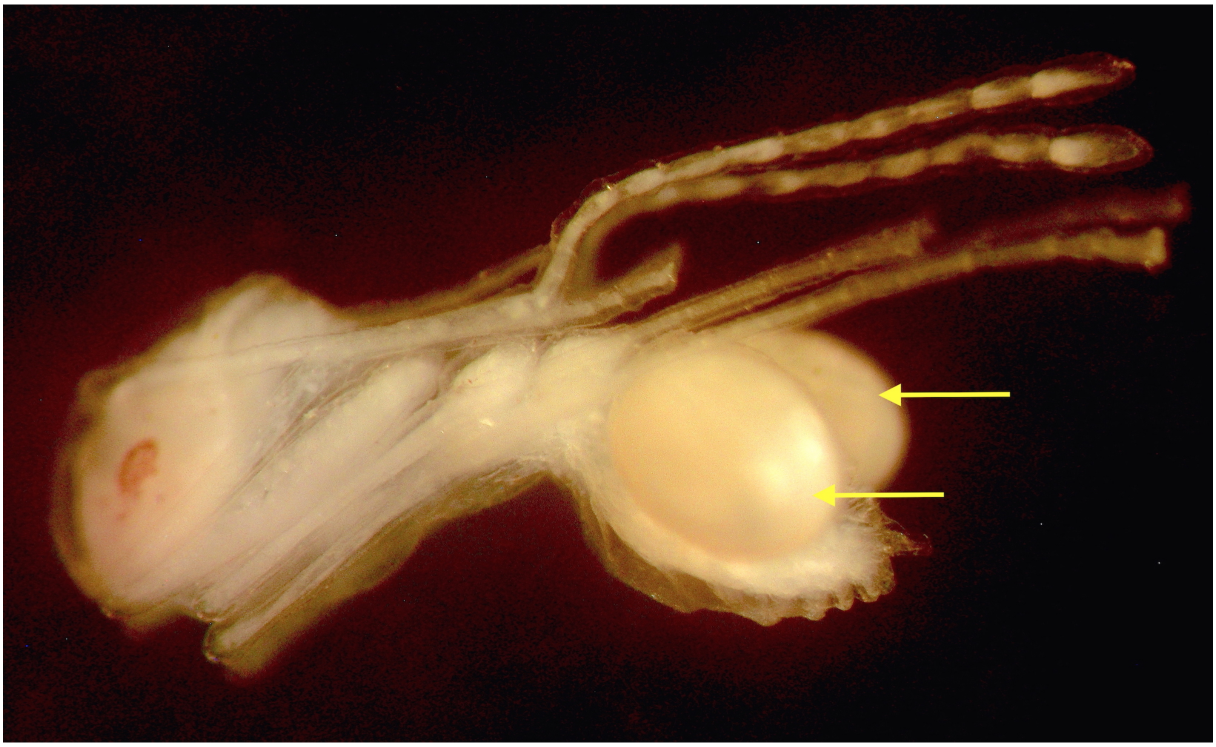

Table S1. Host targets of the different developmental stages of *Macrodinychus multispinosus*. Developmental stages and host caste targeted in parasitized colony samples of *Paratrechina longic*ornis in three localities of southern Quintana Roo, Mexico.

Locality	Date	Parasitized worker pupae			Parasitized male pupae	No. of host pupae	No. of parasitized host pupae
		Larvae	Protonymphs	Deutonymphs + adults	Deutonymphs + adults		
Chetumal	09/09/14	0	0	3	0	13	3
Laguna Guerrero	11/01/14	0	1	4	0	268	5
	01/24/15	2	92	283	0	1596	377
	02/01/15	2	0	0	0	30	2
	06/07/15	6	3	4	0	178	13
	07/27/15	0	511	705	3	1597	1219
Mahahual	09/27/15	0	1	9	0	1330	10
	09/27/15	0	0	1	0	285	1
Total		10	608	1009	3	5297	1630
